# Supplementary material for: Insights into mobile health application market via a content analysis of marketplace data with machine learning
Source: PLoS One. 2021 Jan 6;16(1):e0244302. doi: 10.1371/journal.pone.0244302 (PMC7787530; doi:10.1371/journal.pone.0244302)
Supplement: Attachment — Submitted filename: Response to Reviewers.docx [file pone.0244302.s001.docx]

Reviewer #1: This manuscript presents an investigation on the current landscape of smartphone apps targeting to improve and sustain health and well-being. This study contributes to the current knowledge on m-health application use by reviewing the mobile health applications using content analysis and machine learning algorithms. Although the approach is good, however it require major improvements in order to be considered for acceptance.

1. Overall presentation and structure of the paper is not good, Please consider the following comments:
a. Proper alignment (on right margin) is required.

*Author(s): Thank you for highlighting this issue, the text is properly aligned.*

b. The manuscript has many uneven-sized paragraphs, some are very small and other are too long. Please be consistent in that and make apparently same size of paragraphs, while not disturbing the flow of concepts.

*Author(s): Several improvements were made throughout the text to improve the readability, formatting and paragraph sizes.*

c. On page 4. Line no. 14, 16, 18, 20, 22, 24, and page 5, line 1:- Please start sentence with the capital latter for each starting word.

*Author(s): Thank you, the problems are corrected.*

d. Paper has many punctuation, typo, and grammar mistakes, please proofread it carefully.

*Author(s): The paper was proofread by a native speaker to rectify typos and grammar mistakes.*

e. Conclusion must be revised. Please write a concise conclusion. Also add future directions. The shortcoming and other relevant details can be added into a separate section of discussion with more details.

*Author(s): We have revised the Conclusion and removed the irrelevant section to incorporate related future research directions.*

2. Page 9, Line no. 17 and Page 11, line 3 to 11 :- Please provide references to justify your statements.

*Author(s): Reference(s) has been amended to the related section(s).*

3. Figures are not clear, Please improve image quality and its description in the text must be detailed. Figure 2 is not acceptable, please redraw it.

*Author(s): The figure has been completely changed and redrawn.*

4. Please give more details to improve proposed methodology for reader’s understanding. You may add a proposed Algorithm/ flow chart for the ease of the readers.

*Author(s) An overall model has been added as Figure 2 to be clearer about the model used in the analysis. Moreover, more details on the analysis method and the algorithms are amended into the paper.*

5. In table 2, column 3, why there is an extra sub-column. In table 3, column 3. Please use 0 (zero) before decimal point, for example, instead of writing “.6125” please write as “0.6125” throughout for readability.

*Author(s): The extra column has been removed and 0 was amended before decimal point(s).*

6. Please add a table for different machine learning algorithm output/result comparison in data analysis and result section.

*Authors: The results and performances of each algorithm are presented on Table 4. Yet, a figure has been amended into the relevant section for better visual communication. After the first run of analysis, an extra ANN analysis was performed, and the results are displayed on Table 5. This part has been made clearer in the manuscript. Thank you for the warning.*

7. Overall, all information (apps data, algorithms, results, etc.) are in textual format, Please rewrite using technical details.

*Author(s): Formulas and figures were amended into the paper to make it easier to understand the logic and mathematical foundations of each algorithm applied.*

8. Please make graphs to augment the result analysis.

*Author(s): A pie chart has been added in the discussion section to make final results clearer. Moreover, a chart depicting the performance of each machine learning algorithm was amended to the text*

9. References are too old, please add some latest references.

*Author(s): Six new studies published in the last two years are amended into the text.*

- Agnihothri, S., Cui, L., Delasay, M., & Rajan, B. (2020). The value of mHealth for managing chronic conditions. *Health Care Management Science*, 23, 185–202. https://doi.org/10.1007/s10729-018-9458-2
- Bettiga, D., Lamberti, L., & Lettieri, E. (2020). Individuals’ adoption of smart technologies for preventive health care: a structural equation modeling approach. *Health Care Management Science*, 23, 203–214. https://doi.org/10.1007/s10729-019-09468-2
- Davalbhakta, S., Advani, S., Kumar, S., Agarwal, V., Bhoyar, S., Fedirko, E., Misra, D. P., Goel, A., Gupta, L., & Agarwal, V. (2020). A Systematic Review of Smartphone Applications Available for Corona Virus Disease 2019 (COVID19) and the Assessment of their Quality Using the Mobile Application Rating Scale (MARS). *Journal of Medical Systems*, 44(9). https://doi.org/10.1007/s10916-020-01633-3
- Levine, D. M., Co, Z., Newmark, L. P., Groisser, A. R., Holmgren, A. J., Haas, J. S., & Bates, D. W. (2020). Design and testing of a mobile health application rating tool. *Npj Digital Medicine*, 3(1). https://doi.org/10.1038/s41746-020-0268-9
- Rowland, S. P., Fitzgerald, J. E., Holme, T., Powell, J., & McGregor, A. (2020). What is the clinical value of mHealth for patients? *Npj Digital Medicine*, 3(1). <https://doi.org/10.1038/s41746-019-0206-x>
- Zhang, Y., Liu, C., Luo, S., Xie, Y., Liu, F., Li, X., & Zhou, Z. (2019). Factors influencing patients’ intention to use diabetes management apps based on an extended unified theory of acceptance and use of technology model: Web-based survey. *Journal of Medical Internet Research*, 21(8), 1–17. https://doi.org/10.2196/15023

Reviewer #2: The manuscript presents the analysis of mobile health application market and use machine learning models to predict the download behavior of these apps. Following are points which needs to be considered:
1. The machine learning models used in the study are supervised learning algorithms. How the authors labeled the data, for the models?

*Author(s): For classification the total number of downloads has been used as the class variable. This part has been made clearer in the manuscript. Thank you for highlighting this issue.*

2. What kind of metrics/ qualitative parameters have been extracted from the raw data to feed into the machine learning model for prediction?

*Author(s): A detailed table,* *Table 1 Code Sheet, presents the metrics/ parameters that have been extracted and fed into the machine learning models. This has been made clearer in the manuscript, thank you for pointing this out.*

3. How the authors choose the best factors that influence or effect the download behavior? What was the criteria for choosing the factors?

*Author(s): All the relevant data available on marketplaces regarding each app was put into consideration. This study focuses on the features and keywords that can be extracted from the textual data available in mobile app descriptions as well. Consequently, studies on mobile app acceptance were used as guidelines yet the machine learning algorithms provided distinct keywords related to the download behavior and user scores that were not known to the researchers beforehand. –*

4. The results and analysis section need some more details.

*Author(s): A new table and a chart has been amended into the results section. Moreover, several formulas and a chart has been added to the analysis section to make the algorithms and the analysis methodology easier to understand. The results section is also expanded using new graphs and several amendments to the text.*

5. English language needs proper revisions

*Author(s): The paper was proofread by a native speaker to rectify typos and grammar mistakes.*
