# Supplementary material for: Insights into mobile health application market via a content analysis of marketplace data with machine learning
Source: PLoS One. 2021 Jan 6;16(1):e0244302. doi: 10.1371/journal.pone.0244302 (PMC7787530; doi:10.1371/journal.pone.0244302)
Supplement: Attachment — Submitted filename: Response to Reviewers R2.docx [file pone.0244302.s002.docx]

**Reviewer #1:** I am Happy that the comments are addressed carefully, however, still there are few minor punctuation mistakes that me be addressed:
1. Please use a comma "," after e.g. as e.g., throughout.

***Authors:*** *The authors thank the reviewer the comments and suggestions that definitely helped in improving the manuscript. This minor issue has been settled and a comma was added after `e.g.` throughout the manuscript.*

**Reviewer #2:** Most of the revision prompted by the comments has been incorporated and no further explanation is required.

***Authors:*** *The authors thank the reviewer for the valuable input that helped in improving the manuscript.*

**Additional Editor Comments:**

Please carefully check the paper for English language mistakes including punctuation, typos, etc. I would recommend getting the paper checked by a native speaker.

*The manuscript has been proofread by a native speaker via ‘getproofed’.*
